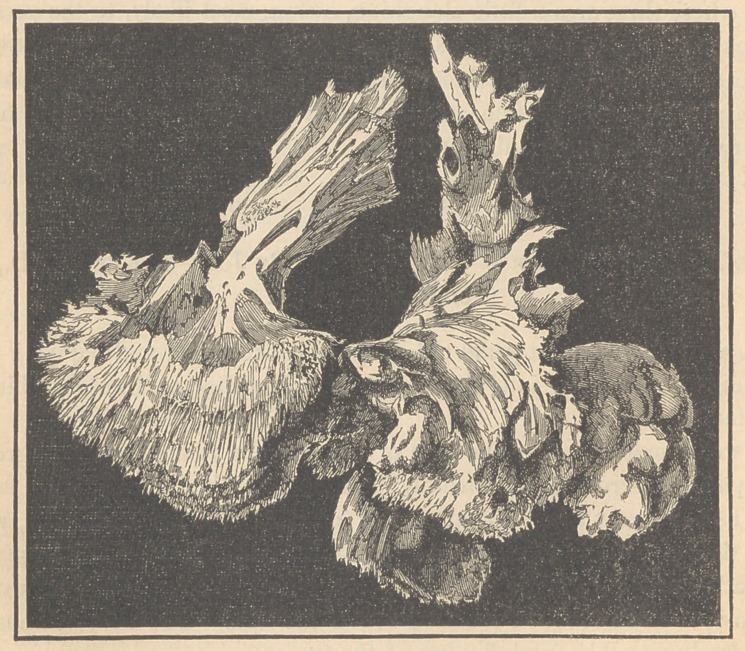# Bony Tumor of the Scrotum

**Published:** 1858-01

**Authors:** John G. Kerr

**Affiliations:** Late of Canton, China


ART. X.—*Bony Tumor of the Scrotum.* By JOHN G. KERR, M.D., late of Canton, China.

A MAN twenty-eight years old, of good constitution, and enjoying good health, was admitted into the Ophthalmic Hospital at Canton, China, in September, 1856.

Twenty months before the time of admission, a tumor began to grow in the patient's scrotum. It steadily increased, and had now attained such a size as to be very inconvenient, and to interfere with his occupation, which was that of a laborer in the country. The tumor was about as large as an infant's head, very hard and dense, and seemed to the touch almost like a mass of stone or wood inclosed within the skin. The skin was healthy, and freely movable over all parts of the tumor.

At my request the operation for its removal was performed by Dr. Geo. B. Newton, Assistant-surgeon of H. B. M. brig "Bittern," who had rendered me important assistance in several operations. The patient was placed under the influence of chloroform, and the tumor skilfully dissected away. The left testicle was carried down before the tumor, and was removed with it. Both testicles were healthy, excepting a slight hydrocele.

The weight of the tumor was five pounds, and was found to consist of numerous cartilaginous lobes of various sizes, densely compacted together with cellular tissue, in which large quantities of bone were deposited. When macerated and cleaned, it presented somewhat the appearance of a coral formation, springing from an irregular semicircular base. Numerous spiculæ of bone were scattered throughout the tumor where apparently separate points of ossification had been established in the different lobules. So numerous were these spiculæ that the knife could scarcely be put into the tumor without touching them.

A part of the bone has a compact outer plate, but the greater portion is spongy and cellular, and composed of radiating spiculæ attached to a firmer base. The process of ossification was interrupted by the operation, and as the tumor had not ceased growing, it is likely that the anomalous formation would have continued to enlarge indefinitely if it had not been removed.

The above figure represents one-half of the bony deposit extracted from the mass by maceration, and now in the possession of Dr. T. G. Richardson, of the Pennsylvania Medical College.

No adequate cause could be discovered for the development of such a tumor in such a place. The patient knew of no injury he had received in the parts. Not only was his general health good, but no alteration of the tissues of the scrotum had taken place.

[A subsequent microscopical examination of the specimen proved it to be genuine bone.—EDS.]

## Figures and Tables

**Figure f1:**